# Opioid Prescribing Rates in Nonmetropolitan and Metropolitan Counties Among Primary Care Providers Using an Electronic Health Record System — United States, 2014–2017

**DOI:** 10.15585/mmwr.mm6802a1

**Published:** 2019-01-18

**Authors:** Macarena C. García, Charles M. Heilig, Scott H. Lee, Mark Faul, Gery Guy, Michael F. Iademarco, Katherine Hempstead, Dorrie Raymond, Josh Gray

**Affiliations:** ^1^Center for Surveillance, Epidemiology, and Laboratory Services, CDC; ^2^National Center for Injury Prevention and Control, CDC; ^3^Robert Wood Johnson Foundation, Princeton, New Jersey; ^4^Athenahealth, AthenaResearch, Watertown, Massachusetts.

Drug overdose is the leading cause of unintentional injury-associated death in the United States. Among 70,237 fatal drug overdoses in 2017, prescription opioids were involved in 17,029 (24.2%) (*1*). Higher rates of opioid-related deaths have been recorded in nonmetropolitan (rural) areas (*2*). In 2017, 14 rural counties were among the 15 counties with the highest opioid prescribing rates.* Higher opioid prescribing rates put patients at risk for addiction and overdose (*3*). Using deidentified data from the Athenahealth electronic health record (EHR) system, opioid prescribing rates among 31,422 primary care providers^†^ in the United States were analyzed to evaluate trends from January 2014 to March 2017. This analysis assessed how prescribing practices varied among six urban-rural classification categories of counties, before and after the March 2016 release of CDC’s *Guideline for Prescribing Opioids for Chronic Pain* (Guideline) (*4*). Patients in noncore (the most rural) counties had an 87% higher chance of receiving an opioid prescription compared with persons in large central metropolitan counties during the study period. Across all six county groups, the odds of receiving an opioid prescription decreased significantly after March 2016. This decrease followed a flat trend during the preceding period in micropolitan and large central metropolitan county groups; in contrast, the decrease continued previous downward trends in the other four county groups. Data from EHRs can effectively supplement traditional surveillance methods for monitoring trends in opioid prescribing and other areas of public health importance, with minimal lag time under ideal conditions. As less densely populated areas appear to indicate both substantial progress in decreasing opioid prescribing and ongoing need for reduction, community health care practices and intervention programs must continue to be tailored to community characteristics.

Athenahealth is a commercial vendor and developer of cloud-based practice management and EHR systems for physician practices and hospitals. Approximately 100,000 health providers, serving about 86 million patients in the United States, use Athenahealth’s applications. This retrospective study used deidentified Athenahealth EHR prescription data from 31,422 primary health care providers serving approximately 17 million patients. Patient-level data were aggregated by week over the 166 weeks from January 5, 2014, through March 11, 2017. For each week during which a patient had at least one Athenahealth record, that patient contributed one patient-week to this analysis. For each patient-week, it was noted whether primary care providers using Athenahealth’s EHR system prescribed one or more opioids (Supplementary Table 1, https://stacks.cdc.gov/view/cdc/61743).^§^ Percentage of patient-weeks during which an opioid prescription was written was considered equivalent to the percentage of patients receiving an opioid prescription during that time.

For comparisons over time, data were divided into three periods. Period 1 comprises 52 weeks from January 5, 2014, through January 3, 2015; period 2 includes the next 63 weeks, ending March 19, 2016; and period 3 covers the final 51 weeks, through March 11, 2017. The first cutpoint allows comparisons between the first and second years’ data, and the second cutpoint supports comparisons before and after the publication of the CDC Guideline. For comparison by population density, data were stratified by providers’ counties according to CDC’s National Center for Health Statistics urban-rural classification scheme.^¶^ From most to least densely populated, the six categories include large central metropolitan, large fringe metropolitan, medium metropolitan, small metropolitan, micropolitan, and noncore counties.

This analysis includes three components. First, the period-specific percentage of patients with opioid prescriptions was estimated empirically and with seasonal adjustment using logistic regression. Second, smooth temporal trends were statistically separated from annual seasonal components using locally weighted regression (*5*). Third, to quantify the period-specific annual rate of increase or decrease in prescribing rates, a second logistic regression model estimated the seasonally adjusted annual percent change (APC) in the odds of receiving an opioid prescription. Statistical software was used for all analyses; statistical tests and confidence intervals (CIs) are presented as simultaneous procedures adjusted for multiple comparisons.

Overall, 128,194,491 patient-weeks of data are included in the analysis; at least one opioid was prescribed during 8,810,237 (6.9%) of these patient-weeks, decreasing from 7.4% during period 1 to 6.4% during period 3 (Table 1) (Supplementary Table 2, https://stacks.cdc.gov/view/cdc/61744). Buprenorphine prescribed for pain and opioid use disorder treatment represented only 0.02% of all opioid prescriptions. By county classification, the overall percentage of patients with opioid prescriptions ranged from 5.2% in large central metropolitan counties to 9.6% in noncore counties during the study period. Patients in noncore counties had an 87% higher chance of receiving an opioid prescription than did patients in large central metropolitan areas during the study period.

**TABLE 1:** Number and percentage of patient-weeks with at least one opioid prescription — Athenahealth, United States, January 2014–March 2017

Urban-rural category*	No. of patient-weeks	No. receiving opioid prescription	Percentage receiving opioid prescription			
			Overall	Period 1^†^	Period 2^†^	Period 3^†^
Noncore	8,979,403	864,364	9.6	10.3	9.9	9.0
Micropolitan	16,342,824	1,532,747	9.4	9.4	9.6	9.1
Small metro	18,860,569	1,443,246	7.7	8.0	7.7	7.4
Medium metro	32,045,592	2,158,111	6.7	7.3	6.9	6.2
Large fringe metro	31,430,958	1,753,802	5.6	6.4	5.8	5.0
Large central metro	20,535,145	1,057,967	5.2	5.4	5.2	5.0
**All counties**	**128,194,491**	**8,810,237**	**6.9**	**7.4**	**7.0**	**6.4**

* National Center for Health Statistics urban-rural classification scheme for counties. https://www.cdc.gov/nchs/data_access/urban_rural.htm.

^†^ Period 1: January 5, 2014–January 3, 2015; period 2: January 4, 2015–March 19, 2016; period 3: March 20, 2016–March 11, 2017. Period-specific percentages are based on raw counts rather than statistical models.

The lowest period-specific percentages of patient-weeks with an opioid prescription occurred in large central metropolitan counties (5.0%–5.4%) (p<0.001, multiplicity-adjusted Wald tests), except during period 3, when percentages in large metropolitan counties (5.0%) were the same as those in large fringe metropolitan counties (5.0%) (Supplementary Table 2, https://stacks.cdc.gov/view/cdc/61744). In contrast, the highest period-specific percentages (9.0%–10.3%) were in noncore counties (p<0.02), except in period 3, when percentages in noncore counties (9.0%) were similar to those in micropolitan counties (9.1%). Across metropolitan and nonmetropolitan categories, all percentages of weeks with an opioid prescription during period 2 were significantly different from those in period 1, and percentages in period 3 differed significantly from those in period 2 (p<0.003).

Visual inspection of the prescribing trends by urban-rural status and by period revealed patterns in both the raw (Supplementary Figure 1, https://stacks.cdc.gov/view/cdc/61741) and seasonally adjusted (Supplementary Figure 2, https://stacks.cdc.gov/view/cdc/61742) data. During period 1, before release of the CDC Guideline, the odds of receiving an opioid prescription increased 6.4% per year in noncore counties (95% multiplicity-adjusted Wald CI = 2.1–10.8), and 9.7% per year in micropolitan counties (95% CI = 6.5–13.0) (Table 2) (Figure). During period 3, after release of the CDC Guideline, the odds of receiving an opioid prescription decreased significantly in all county groups. Comparing trends between periods, the APC increased in large central metropolitan counties in period 2 compared with period 1 (p<0.001) and decreased between periods 2 and 3 (p<0.001). In the other five urban-rural categories, the APC decreased in period 2 compared with period 1 (p<0.02); among these five groups, only micropolitan counties experienced a significant decrease in APC between periods 2 and 3 (p<0.001).

**TABLE 2:** Annual percent change (APC) in odds of receiving at least one opioid prescription — Athenahealth, United States, January 2014–March 2017

Urban-rural category*	Period 1^†^	Period 2^†^	Period 3^†^	p-value (direction of change)^§^	
	APC (95% CI)	APC (95% CI)	APC (95% CI)	Period 1 versus period 2	Period 2 versus period 3
Noncore	6.4 (2.1 to 10.8)^¶^	-10.1 (-12.2 to -8.0)^¶^	-7.5 (-10.7 to -4.2)^¶^	<0.001 (decrease)	0.713 (—)
Micropolitan	9.7 (6.5 to 13.0)^¶^	-0.8 (-2.6 to 0.9)	-13.3 (-15.6 to -10.9)^¶^	<0.001 (decrease)	<0.001 (decrease)
Small metro	0.2 (-2.8 to 3.2)	-4.5 (-6.2 to -2.7)^¶^	-5.8 (-8.4 to -3.2)^¶^	0.013 (decrease)	0.977 (—)
Medium metro	-2.5 (-4.8 to -0.1)**	-8.7 (-10.1 to -7.4)^¶^	-9.2 (-11.2 to -7.2)^¶^	<0.001 (decrease)	0.999 (—)
Large fringe metro	-2.0 (-4.7 to 0.8)	-14.9 (-16.2 to -13.5)^¶^	-13.1 (-15.1 to -10.9)^¶^	<0.001 (decrease)	0.616 (—)
Large central metro	-9.9 (-13.2 to -6.4)^¶^	1.8 (-0.3 to 3.9)	-11.7 (-14.3 to -8.9)^¶^	<0.001 (increase)	<0.001 (decrease)
**All counties**	**-1.4 (-10.6 to 8.8)**	**-8.2 (-13.3 to -2.7)^††^**	**-10.4 (-17.9 to -2.2) ^††^**	**0.371 (—)**	**0.856 (—)**

**Abbreviation:** CI = confidence interval.

* National Center for Health Statistics urban-rural classification scheme for counties. https://www.cdc.gov/nchs/data_access/urban_rural.htm.

^†^ Period 1: January 5, 2014–January 3, 2015; period 2: January 4, 2015–March 19, 2016; period 3: March 20, 2016–March 11, 2017.

^§^ p-values from multiplicity-adjusted Wald tests; (—) indicates a nonsignificant difference (p>0.05) between APCs in adjacent periods.

^¶^ p<0.001.

** p<0.05.

^††^ p<0.01.

**FIGURE:**
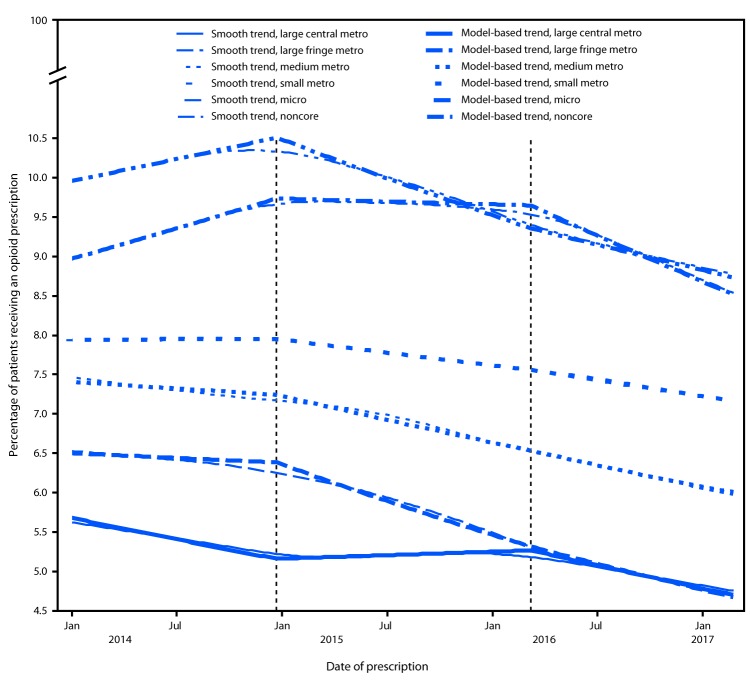
Model-based trends in percentage of patient-weeks with at least one opioid prescription, by urban-rural category — Athenahealth, United States, January 2014–March 2017

## Discussion

Throughout the analysis period, opioid prescribing rates by primary care providers were significantly higher in nonmetropolitan counties than in metropolitan counties. Whereas the prescribing rate increased from January 2014 through January 2015 (period 1) in both micropolitan and noncore counties, those trends halted, and rates became flat or declined through mid-March 2016 (period 2). Trends in all other urban-rural categories were flat or decreasing over the same two periods. The odds of a patient receiving an opioid prescription decreased in all urban-rural county groups after the March 2016 publication of the CDC Guideline. Those trends represented significant decreases in the micropolitan and large central metropolitan categories. In the other four county groups, however, the significant decreases after March 2016 represented a continuation of previously decreasing trends.

Higher odds of opioid prescribing in nonmetropolitan counties might be attributed in part to prescription drug use and misuse at an earlier age as well as higher prevalences of chronic pain among persons living in rural areas (*6*,*7*). Nonmetropolitan counties also tend to have larger populations of older adults who have higher prevalences of conditions associated with pain (*6*). Opioid prescribing in rural (nonmetropolitan) areas is strongly influenced by providers’ individual relationships with their patients (*8*), and can be inconsistent with opioid prescribing guidelines. As well, access to medication-assisted treatment facilities and alternative therapies are limited in rural areas (*8*). Variations in the implementation of state-run prescription drug monitoring programs and state-based laws (*9*), such as the regulation of pain-management clinics, might also differ in urban and rural communities.

Despite reductions in opioid prescribing in recent years (*1*), opioid-involved overdose death rates have increased, largely driven by heroin and illicitly manufactured fentanyl (*2*). Many persons who self-report heroin use have a history of misusing prescription opioids (*10*). Addressing prescription opioid use is an important step in curbing opioid-involved overdose deaths. Interventions such as using Prescription Drug Monitoring Programs and practices that align with evidence-based adoption of the CDC Guideline can improve prescribing decisions.** The Guideline can help providers and patients weigh the benefits and risks of prescribing opioids according to best available evidence and individual patient needs (*4*). This study demonstrates that data from EHRs can effectively supplement traditional surveillance methods for monitoring trends in opioid prescribing and other areas of public health importance. The lag between the collection of the data and this analysis could potentially be reduced to a matter of weeks with optimized workflows.

The findings in this report are subject to at least three limitations. First, the conclusions drawn from the records provided by Athenahealth might not be generalizable to all patients in primary care. Second, although the data include all patients with an opioid prescription, they do not include other characteristics of each prescription, including indication (e.g., chronic versus acute pain or opioid use disorder treated with buprenorphine [although this drug accounted for a small fraction of all opioids prescribed]) and whether prescriptions were filled and taken as prescribed. Finally, this analysis does not account for differing demographic profiles across counties, such as age distributions and payer types, which could be confounded by population density in its association with opioid prescribing rates.

The percentage of patients who received an opioid prescription was lower in more densely populated counties than among less populated rural counties; however, all areas, including rural counties, experienced substantial decreases in prescribing over time. As less densely populated areas appear to indicate both substantial progress in decreasing opioid prescribing and ongoing need for reduction, community health care practices and intervention programs must continue to be tailored to community characteristics.

**Summary**

**What is already known about this topic?**

Opioid prescribing rates vary by county urbanization level and are declining overall.

**What is added by this report?**

Analysis of patient opioid prescription data from a national electronic health record vendor during 2014–2017 found that the percentage of patients prescribed an opioid was higher in rural than in urban areas. Significant decreases in opioid prescribing occurred across all urban-rural categories after the March 2016 release of the *CDC Guideline for Prescribing Opioids for Chronic Pain.*

**What are the implications for public health practice?**

As less densely populated areas indicate both progress in decreasing opioid prescribing and need for ongoing reduction, tailoring community health care practices and intervention programs to community characteristics will remain important.

## Footnotes

* U.S. Opioid Prescribing Rate Maps. https://www.cdc.gov/drugoverdose/maps/rxrate-maps.html.

† Primary care providers in an ambulatory setting; limited to family medicine, family practice, or general practice, or providers who have an internal medicine specialty with no subspecialty. Nurse practitioners and physician assistants are included among primary care providers.

§ Short and long acting opioid drugs in this study included buprenorphine, butorphanol, codeine, dihydrocodeine, fentanyl, hydrocodone, hydromorphone, levorphanol, meperidine, methadone, morphine, naltrexone, nalbuphine, naloxone, oxycodone, oxymorphone, pentazocine, propoxyphene, tapentadol, and tramadol. The study does not count cough and cold medications containing opioids.

¶ https://www.cdc.gov/nchs/data_access/urban_rural.htm; https://www.cdc.gov/nchs/data/series/sr_02/sr02_166.pdf.

** https://www.cdc.gov/drugoverdose/pdf/prescribing/CDC-DUIP-QualityImprovementAndCareCoordination-508.pdf.
